# On-demand Hydrogen Production from Organosilanes at Ambient Temperature Using Heterogeneous Gold Catalysts

**DOI:** 10.1038/srep37682

**Published:** 2016-11-24

**Authors:** Takato Mitsudome, Teppei Urayama, Taizo Kiyohiro, Zen Maeno, Tomoo Mizugaki, Koichiro Jitsukawa, Kiyotomi Kaneda

**Affiliations:** 1 Department of Materials Engineering Science, Graduate School of Engineering Science, Osaka University, 1-3 Machikaneyama, Toyonaka, Osaka 560-8531, Japan; 2 Research Center for Solar Energy Chemistry, Osaka University, 1-3 Machikaneyama, Toyonaka, Osaka 560-8531, Japan

## Abstract

An environmentally friendly (“green”), H_2_-generation system was developed that involved hydrolytic oxidation of inexpensive organosilanes as hydrogen storage materials with newly developed heterogeneous gold nanoparticle catalysts. The gold catalyst functioned well at ambient temperature under aerobic conditions, providing efficient production of pure H_2_. The newly developed size-selective gold nanoparticle catalysts could be separated easily from the reaction mixture containing organosilanes, allowing an on/off-switchable H_2_-production by the introduction and removal of the catalyst. This is the first report of an on/off-switchable H_2_-production system employing hydrolytic oxidation of inexpensive organosilanes without requiring additional energy.

Hydrogen (H_2_) is one of the most promising energy carrier alternatives to fossil fuels. However, realizing a H_2_-powered society is not so easy due mainly to problems related to the storage and transportation of H_2_. The efforts to develop efficient and convenient H_2_-generation systems have led to investigations into hydrogen storage materials such as metal hydrides and chemical hydrides. Metal hydrides, such as LiAlH_4_ and AlH_3_, react violently when they contact water at ambient temperature, providing the rapid production of large amounts of pure H_2_^1^,^2^,^3^. However, these reactions are difficult to control and can result in an explosion. Chemical hydrides, such as ammonia-borane^4^,^5^,^6^ and formic acid^7^,^8^, are leading candidates for new hydrogen storage materials because of their hydrogen content and highly efficient catalysts for producing H_2_ from these molecules have been reported^9^,^10^,^11^,^12^,^13^,^14^,^15^,^16^,^17^,^18^,^19^,^20^,^21^,^22^,^23^. However, for effective functioning, these catalysts often require heat energy^9^,^10^,^14^ and anaerobic conditions^11^,^12^,^14^,^15^,^22^. In addition, these reactions can result in toxic ammonia salt and CO by-products, and the co-production of CO_2_ gas from formic acid that requires a separation process using membranes to obtain pure H_2_ gas, which limit their wide utilization for producing H_2_.

The hydrolytic oxidation of organosilanes can be an efficient H_2_-production method. Several organosilanes are relatively air-stable and non-toxic. Additionally, polymethylhydrosiloxane (Me_3_Si(OSiMeH)_n_OSiMe_3_, PMHS) and tetramethyldisiloxane (Me_2_SiHOSiHMe_2_, TMDS) are by-products of the silicon industry and so are cost-effective^24^,^25^, and the resulting silanols are useful as additives for silicon rubber^26^,^27^. Therefore, the development of highly efficient catalysts that promote hydrolytic oxidation of organosilanes and can function at ambient temperature under aerobic conditions may provide efficient production of pure H_2_ without additional energy input. In addition, the use of heterogeneous catalysts should allow easy separation from organosilane, leading to on/off-switching of H_2_-production. Several heterogeneous catalysts for the hydrolytic oxidation of organosilanes have been reported^28^,^29^,^30^,^31^,^32^,^33^,^34^,^35^,^36^,^37^. However, these catalysts were developed for the synthesis of organosilanols, and no attention has been paid for the utilization of heterogeneous catalysts for H_2_-production through hydrolytic oxidation of inexpensive organosilanes such as PMHS and TMDS.

The present report describes the development of an efficient controllable H_2_-production system using hydrolytic oxidation of organosilanes with newly developed heterogeneous gold nanoparticle (AuNP) catalysts at ambient temperature under aerobic conditions. Addition and removal of the heterogeneous AuNP catalyst from the reaction mixture enabled easy on-demand production of pure H_2_ without any additional energy input. In the case of hydrolytic silane-oxidation, the AuNP catalysts exhibited the highest turnover numbers and turnover frequencies of up to 3,333,000 and 77/sec, respectively, that have so far been reported. Moreover, the AuNP catalysts were reusable without loss of activity as demonstrated during recycling experiments.

## Results and Discussion

We recently reported that gold^38^,^39^ and silver^40^ NPs were capable of promoting hydrolytic oxidation of hydrosilanes to silanols. Hydroxyapatite (HAP)-supported AuNPs with a mean diameter of 3 nm (Au/HAP) prepared by a deposition–precipitation method acted as a highly efficient heterogeneous catalyst. The Au/HAP showed high catalytic activity at ambient temperature under aerobic conditions, producing pure H_2_ during the reaction^39^. Thus, we attempted to improve the catalytic activity of Au/HAP by decreasing the size of AuNPs to create more efficient heterogeneous catalysts. The size-control synthesis of AuNPs on HAP was performed using a modification of a previously reported method that used glutathione as a capping reagent for the AuNPs^41^,^42^,^43^. Briefly, glutathione (1.0 mmol) was added to a methanol solution (50 mL) of HAuCl_4_ (0.25 mmol) and stirred for 30 min at 273 K in air. Next, KBH_4_ (1.0 mmol) was added to the solution. After stirring 1 h at 273 K, the solid was collected by centrifugation and the precipitate was re-dispersed in water. Different amounts of HAP were added to this dispersion, followed by stirring for 4 h at r.t. HAP is well known to have high adsorption property for amino acid and protein^44^,^45^, therefore, the glutathione-modified Au nanoparticles would be easily adsorbed on HAP. Actually, the colloidal Au nanoparticles capped with glutathione was easily adsorbed on HAP by simply stirring the colloidal Au nanoparticles solution in the presence of HAP. The mixture was then filtered, washed with deionized water, and dried *in vacuo*. Finally, the solid obtained was calcined at 400 °C in air for 8 h to remove glutathione coordinated to AuNPs, giving Au/HAP-NC. The sulfur in Au/HAP-NC was not detected by elemental analysis, confirming the removal of the glutathione capping reagent. The loading amount of Au X wt% are designated as Au/HAP-NC (X wt%).

**Figure 1:**
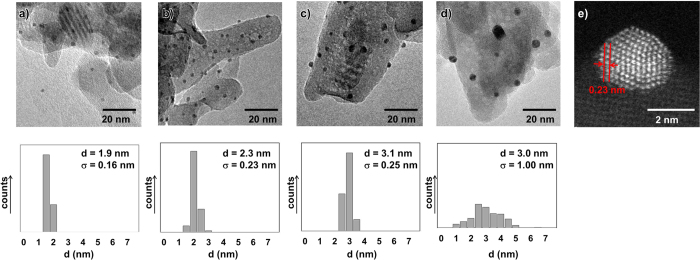
TEM images and corresponding histograms for (**a**) Au/HAP-NC (0.5 wt%); (**b**) Au/HAP-NC (2 wt%); (**c**) Au/HAP-NC (3 wt%); (**d**) Au/HAP. (**e**) ADF-STEM image of Au/HAP-NC (0.5 wt%).

Representative images of Au/HAP-NC obtained by transmission electron microscopy (TEM) are depicted in Fig. 1. Au/HAP-NC showed small AuNPs with a very narrow size distribution. Au/HAP-NC (0.5 wt%), Au/HAP-NC (2 wt%), and Au/HAP-NC (3 wt%) had AuNPs with mean diameters of 1.9 nm, 2.3 nm, and 3.1 nm, respectively (Fig. 1a–c), whereas Au/HAP prepared by the deposition–precipitation method had AuNPs with a mean diameter of 3.0 nm with a relatively broad size distribution (Fig. 1d). The lattice fringe had *d*-spacing attributed to d-spacing Au {111} (Fig. 1e). These results revealed that glutathione allowed the size-selective synthesis of small AuNPs on HAP and that the size of AuNPs can be controlled by the Au loading amounts. Au/HAP-NC was also characterized by Ultraviolet-Visible Absorption Spectroscopy (UV-Vis), X-ray Diffraction (XRD), and X-ray Photoelectron Spectroscopy (XPS) (Supplementary Figures 2–4).

**Figure 2:**
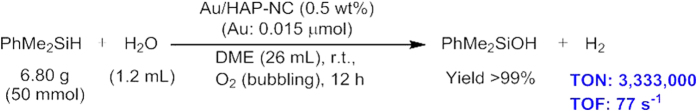
Scale-up reaction using Au/HAP-NC (0.5 wt%).

With the prepared Au/HAP-NC catalysts in hands, the hydrolytic oxidation of dimethylphenylsilane (**1**) as a model substrate was conducted in dimethoxyethane (DME) at ambient temperature in air. Results are shown in Table 1. Notably, Au/HAP-NC (0.5 wt%) efficiently promoted the oxidation, affording dimethylphenylsilanol (**2**) in 99% yield along with the generation of equimolar amounts of H_2_ after 9 min (Table 1, entry 2). The catalytic activity of Au/HAP-NC increased as the size of the AuNPs decreased (entries 1, 4, and 5). The catalytic activity of Au/HAP-NC (0.5 wt%) was much greater than that of previously reported Au/HAP prepared by the deposition–precipitation method (entry 6). Neither bulk Au (non-nanosized Au) nor HAP show any activity (entries 7 and 8), indicating that the AuNPs were the active species. In addition, Au/HAP-NC (0.5 wt%) worked well at scale-up conditions with a lower catalyst loading (0.03 mmol%), giving a turnover number of 3,333,000 and turnover frequency of 77/sec based on the total amount of Au used in the reaction (Fig. 2), both of which were much greater than values previously reported (Supplementary Table 1).

**Figure 3:**
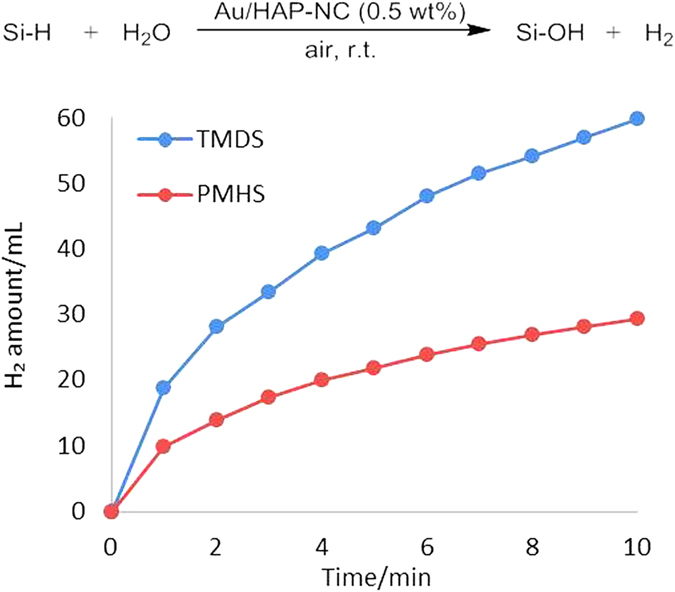
Evolution of H_2_ from TMDS or PMHS using Au/HAP-NC (0.5 wt%). Reaction conditions: Au/HAP-NC (0.5 wt%) (1.0 g, Au: 0.025 mmol), Si-H (10 mmol), DME (5 mL), water (0.6 mL), air.

Next, the catalytic activity of Au/HAP-NC (0.5 wt%) for H_2_-production through the hydrolytic oxidation of PMHS and TMDS was investigated. The time-course for H_2_-production is shown in Fig. 3. Upon addition of Au/HAP-NC (0.5 wt%) to the DME/water solution of PMHS or TMDS at ambient temperature in air, H_2_ gas was efficiently generated with initial production rates of 9.8 and 18.9 mL/min, respectively. When Au/HAP-NC (0.5 wt%) was separated from the reaction mixture by filtration, the H_2_ generation quickly stopped; re-addition of Au/HAP-NC (0.5 wt%) to the filtrate induced re-generation of H_2_. The generation and suppression of H_2_ were repeatable, enabling the on/off-switching of H_2_-production by the introduction and removal of catalyst. The catalytic system of the on/off-switching of H_2_-production was demonstrated in Supplementary Movie 1; the generation and suppression of the H_2_ bubble in response to the introduction and removal of Au/HAP-NC (0.5 wt%) could be observed.

This on/off-switchable H_2_-production system could be applied to a portable hydrogen fuel cell. When the catalyst system (hydrogen generation part) was connected to the power generation part, including a Pt anode, electric power was generated that could be turned on and off at ambient temperature in air atmosphere (Supplementary Movie 2). This is the first report of an on/off-switchable H_2_-production system using the hydrolytic oxidation of inexpensive organosilanes without the need for any additional energy input. The TEM image of the used Au/HAP-NC (0.5 wt%) catalyst showed that the average diameter and size distribution of the AuNPs were similar to those of Au/HAP-NC (0.5 wt%) before use and that no aggregation of AuNPs occurred (Supplementary Figure 1), proving high durability of Au/HAP-NC (0.5 wt%) against aggregation.

In conclusion, a size-controllable and size-selective synthesis of AuNPs on HAP was developed. The Au/HAP-NC (0.5 wt%) catalyst with a mean diameter of 1.9 nm possessed the greatest catalytic activity for the aqueous oxidation of silanes. The high catalytic activity of Au/HAP-NC (0.5 wt%) was applied to H_2_-production from inexpensive PMHS and TMDS as hydrogen storage materials at ambient temperature in air. The solid Au/HAP-NC (0.5 wt%) catalyst could be separated, enabling the on/off-switching of H_2_-production by the introduction and removal of the catalyst. This catalytic system for on/off switchable H_2_-production from organosilanes can contribute to the development of next-generation green hydrogen fuel cells with on-demand H_2_-production.

## Methods

### Synthesis of Au/HAP-NC (X wt%)

Glutathione (1 mmol) was added to a methanol solution (50 mL) of HAuCl_4_ (0.25 mmol) and stirred for 30 min at 273 K in air. Next, a methanol solution of KBH_4_ (1.0 mmol) was added. After stirring 1 h at 273 K, the solid was collected by centrifugation and the precipitate re-dispersed in water (100 mL). Different amounts of HAP were added to the dispersion, followed by stirring for 4 h at r.t. The mixture then was filtered, washed with deionized water, and dried *in vacuo*. Finally, the obtained solid was calcined at 400 °C in air for 8 h to remove the glutathione coordinated to AuNPs, giving Au/HAP-NC. The loading amount of Au X wt% is designated as Au/HAP-NC (X wt%).

### Typical reaction procedure

A typical reaction procedure for oxidation of **1** to **2** using Au/HAP-NC (0.5 wt%) was as follows. The Au/HAP-NC (0.5 wt%) (0.02 g, Au: 0.05 mol%) was placed in a reaction vessel, followed by addition of DME (2 mL), water (0.2 mL), and **1** (1 mmol). The reaction mixture was stirred vigorously at 30 °C under an air atmosphere for 8 min. Then, the Au/HAP-NC (0.5 wt%) was filtered and the yield determined by GC analysis.

### Measurement of H_2_

The amount of H_2_ generated during the reaction was measured using the water displacement method. Qualitative analyses of generated gas were performed by GC-TCD. GC conditions were as follows: thermal conductivity detector (Shimadzu GC-8A); column: molecular sieves 13X (4.0 m); oven temperature: 40 °C; injection and detection temperature: 70 °C; carrier gas: Ar (100 kPa); current: 60 mA; retention time: H_2_ (5.3 min).

## Additional Information

**How to cite this article**: Mitsudome, T. *et al*. On-demand Hydrogen Production from Organosilanes at Ambient Temperature Using Heterogeneous Gold Catalysts. *Sci. Rep.* **6**, 37682; doi: 10.1038/srep37682 (2016).

**Publisher's note:** Springer Nature remains neutral with regard to jurisdictional claims in published maps and institutional affiliations.

## Supplementary Material

Supplementary Information

Supplementary movie 1

Supplementary movie 2

## Figures and Tables

**Table 1:**
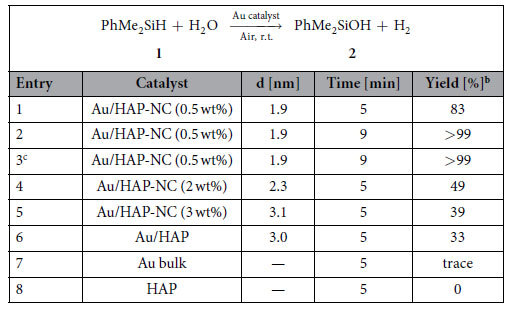
Aqueous oxidation of 1 using Au catalyst^a^.

^a^Reaction conditions: **1** (1 mmol), Au catalyst (0.05 mol%), water (0.2 mL), DME (2 mL).

^b^Determined by GC using internal standard technique.

^c^5th reuse.
