# Supplementary material for: On-demand Hydrogen Production from Organosilanes at Ambient Temperature Using Heterogeneous Gold Catalysts
Source: Sci Rep. 2016 Nov 24;6:37682. doi: 10.1038/srep37682 (PMC5121617; doi:10.1038/srep37682)
Supplement: Supplementary Information [file srep37682-s1.pdf]

Supplementary Information

On-demand Hydrogen Production from Organosilanes at Ambient Temperature Using Heterogeneous Gold Catalysts

Takato Mitsudome[1], Teppei Urayama[1], Taizo Kiyohiro[1], Zen Maeno[1], Tomoo Mizugaki[1], Koichiro Jitsukawa[1], Kiyotomi Kaneda[1,2,]*

[1] Department of Materials Engineering Science, Graduate School of Engineering Science, Osaka University, 1-3 Machikaneyama, Toyonaka, Osaka 560-8531, Japan
Fax: (+81) 6-6850-6260, E-mail: kaneda@cheng.es.osaka-u.ac.jp

[2] Research Center for Solar Energy Chemistry, Osaka University, 1-3, Machikaneyama, Toyonaka, Osaka 560-8531, Japan

1) General

The $HAuCl_4 \cdot xH_2O$ was obtained from Mitsuwa Chemicals Co., Ltd., and HAP (apatite HAP, monoclinic, $Ca_{10}(PO_4)_6(OH)_2$) was purchased from Wako Pure Chemical Co., Ltd. The GC-FID and GC-MS were performed on a Shimadzu GC-2014 instrument equipped with a Unisole-30T column and a GCMS-QP2010 SE instrument equipped with an Inert Cap WAX-HT capillary column (30 m × 0.25 mm i.d., 0.25 μm. Gas-phase analysis was done with an on-line quadrupole mass spectrometer (BELMass-S, BEL Japan, Inc.). The ^{1}H and ^{13}C nuclear magnetic resonance (NMR) spectra were recorded on a JEOL JNM-ESC400 spectrometer and the chemical shifts reported in ppm from TMS as a reference. Transmission electron microscopy observations were obtained using an FEI Tecnai G2 20ST instrument operated at 200 kV. XRD analysis was performed using X'pert PRO (Yamato Scientific Co., Ltd.). UV-Vis spectra were recorded with V-670 (JASCO). XPS spectra were obtained using ESCA-3400HSE (Shimadzu Analytical and Measuring Instruments, Mg-Kα, 10 kV, 20 mA).

2) Synthesis of Au/HAP

Supported gold NPs were synthesized as follows. The HAP (1.0 g) was soaked in 50 mL of an aqueous solution of $HAuCl_4$ (2 mM). After stirring for 2 min, 0.2 mL of aqueous NH_3 (10%) was added and the resulting mixture stirred at room temperature for 12 h in an air atmosphere. The obtained slurry was filtered, washed with deionized water, and then dried at room temperature *in vacuo*. Subsequently, the HAP solid containing Au ions was added to 50 mL of an aqueous solution of KBH_4 (18 mM) and stirred at room temperature for 1 h to yield Au/HAP as a purplish red powder.

3) Characterization of Au/HAP-NC

(a) Au/HAP-NC (0.5 wt%) (fresh)

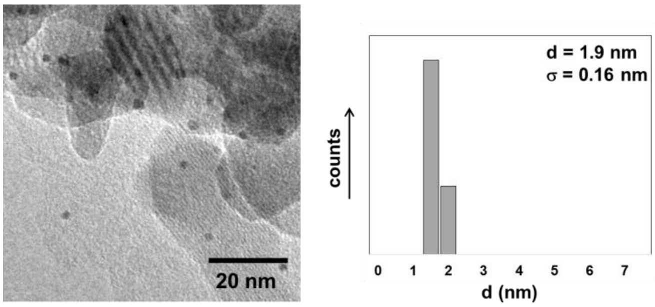

(b) Au/HAP-NC (0.5 wt%) (after reuse experiment)

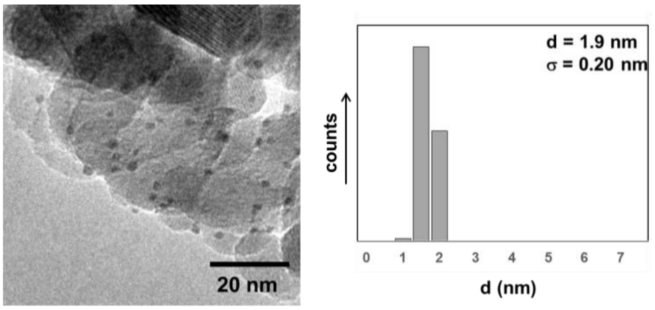

Supplementary Figure 1. TEM images of Au/HAP-NC (0.5 wt%) and its size distribution histogram. (a) Au/HAP-NC (0.5 wt%) (fresh), (b) Au/HAP-NC (0.5 wt%) (after reuse experiment).

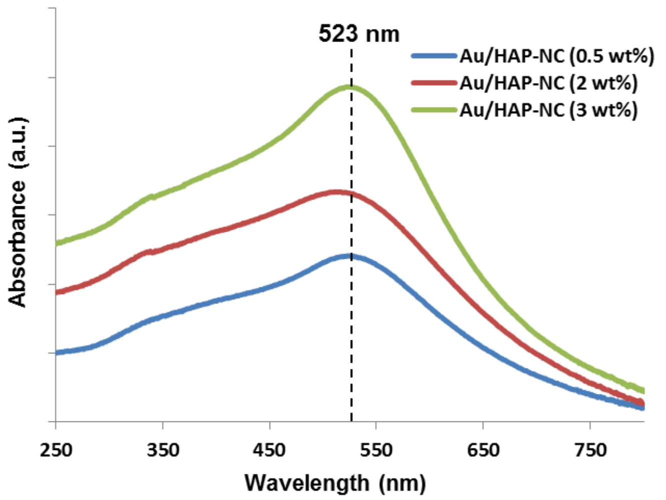

Supplementary Figure 2. DRIFT UV-Vis spectra of Au/HAP-NC catalysts. The surface plasmon resonance absorptions of Au NPs were observed at 523 nm.

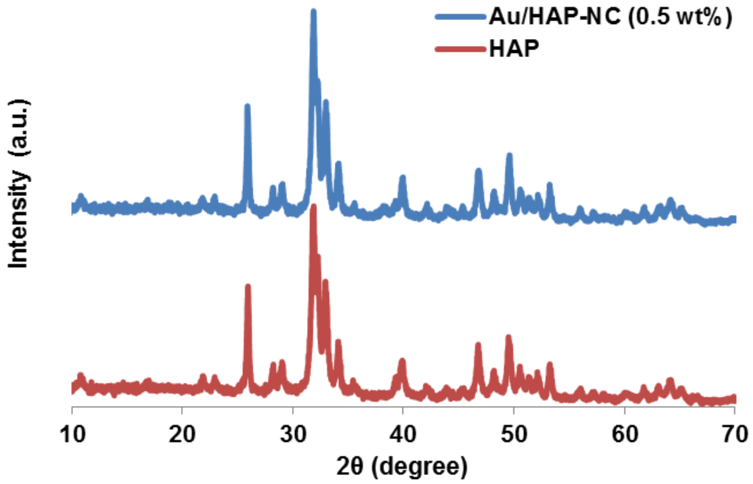

Supplementary Figure 3. XRD measurement of Au/HAP-NC (0.5 wt%) and HAP. The structure of HAP was maintained after immobilizing AuNPs.

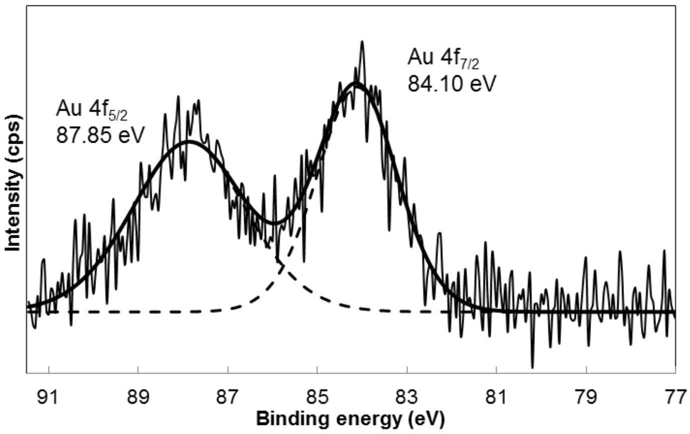

Supplementary Figure 4. XPS spectra of Au/HAP-NC catalysts. The peak of Au $4f_{7/2}$ and $4f_{5/2}$ were located at 84.10 eV and 87.85 eV respectively, indicating that the oxidation state of Au supported on HAP was metallic state (0 valent).

4) Kinetic study

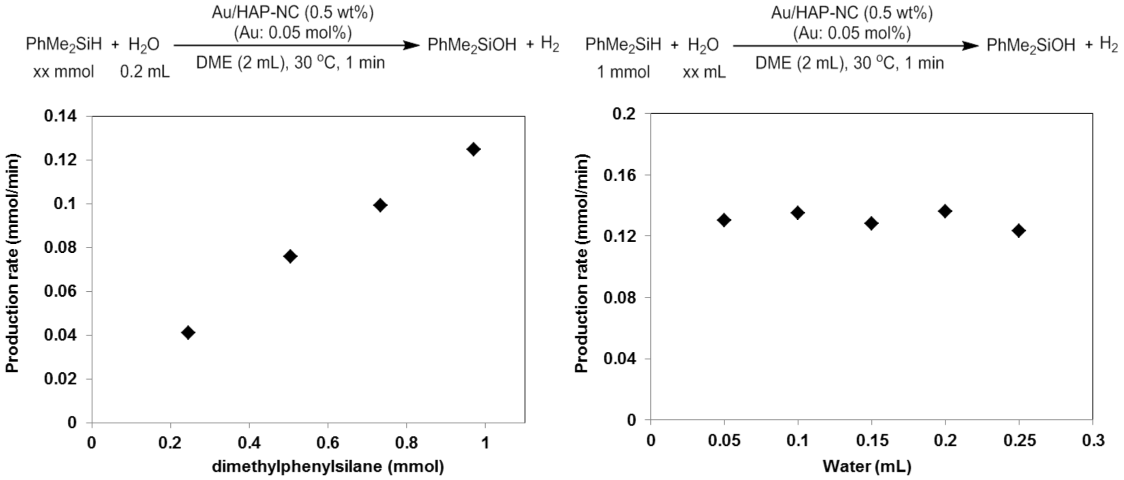

Supplementary Figure 5. Dependency on the concentration of dimethylphenylsilane and the amount of water. The reaction rate was proportional to the amount of hydrosilane and independent of the concentration of water.

5) Large-scale reaction

A large-scale reaction was performed under O_2 bubbling for 12 h. After the oxidation reaction, Au/HAP-NC was filtered and the yield determined by GC analysis. The product was isolated by Kugelrohr distillation.

6) Demonstration of on/off-switching of H_2 production by introduction and removal of Au/HAP-NC (0.5 wt%)

Please see Supplementary Movie 1. Demonstration of on/off-switching of H_2 production by introduction and removal of Au/HAP-NC (0.5 wt%).

Reaction conditions: Au/HAP-NC (0.5 wt%) (1.0 g, Au: 0.025 mmol), TMDS (4.0 g), DME (20 mL), water (1.5 mL), air atmosphere.

7) Utilization of catalytic H_2-generation system for portable fuel cell

Please see Supplementary Movie 2. Utilization of catalytic H_2-generation system for portable fuel cell.

Module cell AF1FC_M was obtained from Aquafairy. Reaction conditions: Au/HAP-NC (0.5 wt%) (0.5 g, Au: 0.0125 mmol), TMDS (4.0 g), DME (20 mL), water (1.5 mL), air atmosphere. This developed system is also applicable to use more environment-friendly solvents ethyl acetate and acetone instead of DME.

8) Comparison with previously reported catalysts

Supplementary Table 1. Comparison with previous reported catalysts

Catalyst	TON	TOF (sec^{-1})	Reference (Ref. X)
Au/HAP-NC (0.5 wt%)	3,333,000	77	This work
Au/HAP	1,230,000	49	Our previous work (39)
Pd/Al_2O_3	20,000	28	J. Park *et al. ChemCatChem* (33)
Pd/C	20,000	28	K. Shimizu *et al. Chem. Eur. J.* (32)
AuCNT nanohybrid	12,000	20	E. Doris *et al. Angew. Chem. Int. Ed.* (31)
$Au_{34}Ag_{28}(PA)_{34}$/Carbon	-	9 (32)[a]	N. Zheng *et al. J. Am. Chem. Soc.* (37)
Au/SiO_2	250	5[a]	T. Zhang et al. *Chem. Commun.* (35)
$TBA_8[Ag_4(\gamma\text{-}H_2SiW_{16}O_{32})_2]$	27,000	4	N. Mizuno *et al. Angew. Chem. Int. Ed.* (34)

[a]Caliculated based on surface metal atoms.

9) Hydrogen production through hydrolytic oxidation of PMHS and TMDS

The generation of equimolar amounts of H_2 through oxidation of PMHS and TMDS was confirmed by GC-TCD analyses. The ^{1}H NMR analyses indicated that the Si-H bonds of these substrates were completely oxidized after hydrogen production (Supplementaly Figures 6 and 7).

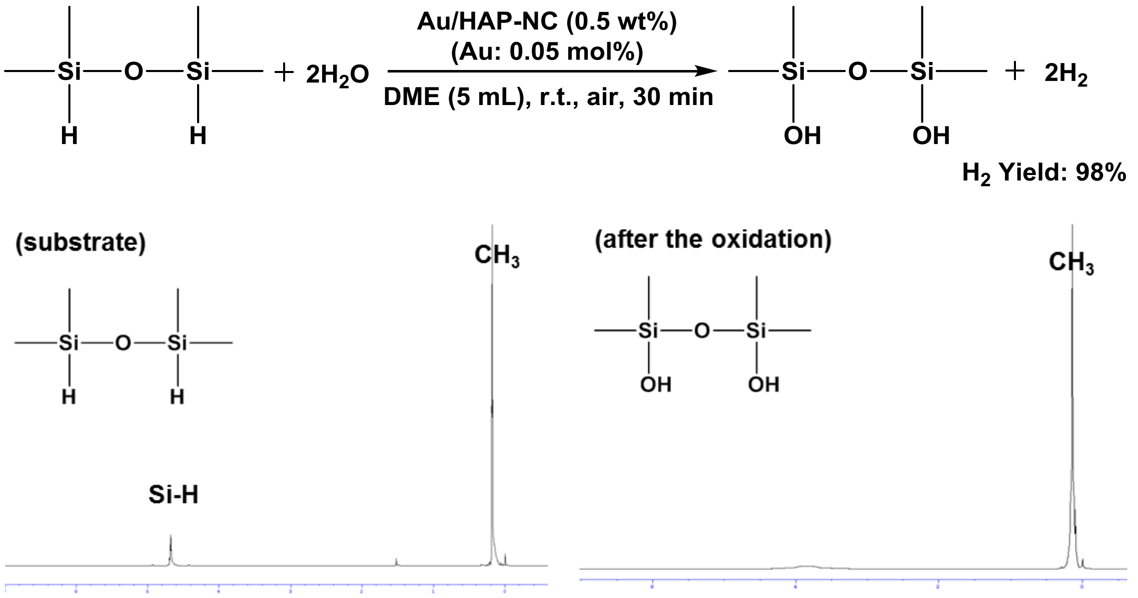

Supplementary Figure 6. Hydrogen production through oxidation of TMDS

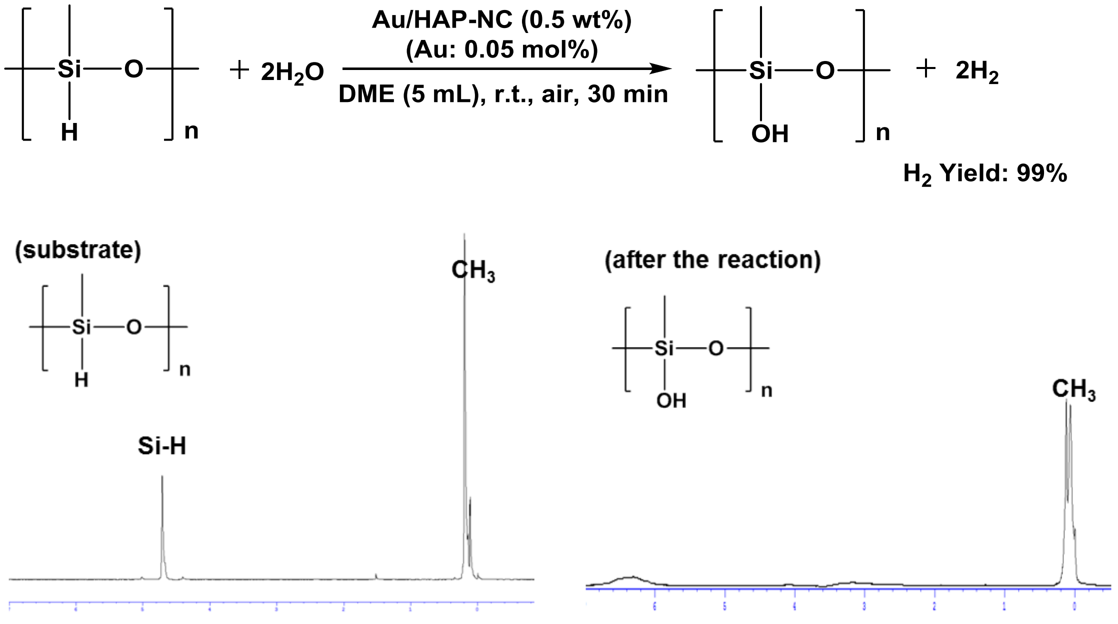

Supplementary Figure 7. Hydrogen production through oxidation of PMHS

10) Product identification

The products were characterized by GC, GC-MS, and NMR. Retention times (GC) and chemical shifts (^{1}H and ^{13}C NMR) of the products agreed with those of authentic samples or previously reported values.

Dimethylphenylsilanol: CAS registry No. [5272-18-4] (Table 1)

^{1}H NMR (400 MHz, $CDCl_3$): δ 7.54-7.62 (m, 2H), 7.32-7.36 (m, 3H), 0.39 ppm (s, 6H).
